# Different Processing Practices and the Frying Life of Refined Canola Oil

**DOI:** 10.3390/foods8110527

**Published:** 2019-10-24

**Authors:** Randy Adjonu, Zhongkai Zhou, Paul D. Prenzler, Jamie Ayton, Christopher L. Blanchard

**Affiliations:** 1 ARC Industrial Transformation Training Centre for Functional Grains, Charles Sturt University, Wagga Wagga, NSW 2650, Australia; zhongkai_zhou@hotmail.com (Z.Z.); pprenzler@csu.edu.au (P.D.P.); cblanchard@csu.edu.au (C.L.B.); 2 Graham Centre for Agricultural Innovations, Charles Sturt University, Wagga Wagga, NSW 2650, Australia; 3 Key Laboratory of Food Nutrition and Safety, Ministry of Education, Tianjin University of Science and Technology, Tianjin 300457, China; 4 School of Food Engineering and Biotechnology, Tianjin University of Science and Technology, Tianjin 300457, China; 5 NSW Department of Primary Industries, Wagga Wagga Agriculture Institute, Wagga Wagga, NSW 2650, Australia; jamie.ayton@dpi.nsw.gov.au

**Keywords:** refined canola oil, tocopherols retention, total polar compounds, frying life, antioxidant activity

## Abstract

Refined expeller-pressed (RCanO-I and RCanO-II) and expeller-pressed and solvent-extracted blended (RCanO-III and RCanO-IV) canola oils were compared to determine the effect of processing (extraction) practice on the frying life of canola oil. Samples were from the 2016/2017 and 2017/2018 production seasons and were used to fry potato chips for 36 to 48 cycles. Frying life was assessed by the total polar compounds, retention of tocopherols, antioxidant activity, and other quality indices. RCanO-II exhibited significantly, the longest frying life as compared with the other three oils and this correlated with tocopherol retention and antioxidant activity (*p* < 0.05). The extraction practice influenced the frying life of canola oil, but this was dependent on other processing practices employed by the individual processors. Variations in initial oil quality dictated the rates of chemical reactions occurring in the oils during frying and influenced oil stability.

## 1. Introduction

Canola oil is an economically important vegetable oil, behind only palm and soybean oil in terms of production and domestic consumption. In recent years, the consumption and use of canola oil have attracted much attention due to the balanced fatty acids composition, i.e., oleic acid (50% to 70%), linoleic acid (15% to 30%), and linolenic acid (5.0% to 14%). Canola oil also contains a low amount of saturated fatty acids (<7%) [1], as compared with other edible oils such as olive, palm, cotton, coconut, and palm kernel oils.

Canola oil is produced by first extracting the crude oil from canola seeds either by expeller pressing only or expeller pressing followed by solvent extraction. The crude oil is chemically or physically refined to remove gums and sediments, heavy metals, pigments, pro-oxidants (e.g., chlorophyll), peroxides, free fatty acids, and other malodorous compounds. The refining process increases the stability and shelf life, ensures a bland taste to facilitate the expansion of the aroma and flavor of the cooked food, and makes the oil safer to handle by elevating the smoke and flash points. Substantial compositional differences exist between oils recovered by different extraction methods. Studies have reported differences in free fatty acids, peroxide value, phosphatides, chlorophyll, tocopherols, phytosterols, and polyphenols for crude canola oil obtained by either of the extraction techniques [1,2]. Generally, solvent extracted oil has higher concentrations of free fatty acids, phosphatides, chlorophyll and is darker in color, which can present refining challenges and increase refining costs. Nevertheless, solvent extracted crude canola oil has been shown to have higher tocopherols and phytosterols content [1,2], and slightly higher oxidative stability than expeller-pressed crude canola oil [2].

These variations in crude oil composition can lead to compositional and functional differences in the refined oil. Warner and Dunlap [3] found that potato chips fried in expeller-pressed, physically refined soybean oil had significantly better sensory (i.e., lower intensity of fishiness) and quality attributes than chips fried in solvent-extracted refined soybean oil. These observed differences were attributed to variations in minor components between the two types of oil. Van Hoed et al. [2], however, indicated that refining produces products with comparable quality. 

Deep frying in heated edible oil is a popular method of food preparation globally [4]. It has been used over many decades to prepare foods owing to the excellent sensory characteristics of fried foods. It is a process involving a complex interplay of moisture, oxygen and heat, coupled with mass and energy transfers between the food and the hot oil. This results in formation of a diverse array of chemical compounds with different polarities, stabilities, and molecular weights [4]. The complex reactions and the compounds formed account for the desirable crispiness, flavor, taste, and golden color of fried foods. However, hydrolysis, oxidation and polymerization reactions of triglycerides during repeated frying cause oil degradation, resulting in off-flavors and accumulation of potentially toxic compounds. Typical oils used for frying include soybean [3,5], corn, hazelnut [5], peanut [6], canola, palm, palm kernel [7,8], extra virgin olive [9], and olive pomace [4] oils. 

Frying studies have focused on oils from different plant sources with marked variations in their fatty acid compositions [7,8,10]; the effect of minor components, tocopherols, sterols, and polyphenols [11]; the type and composition of food [12]; and the frying conditions [13], however, no systematic studies has been done on the effect that different processing practices may have on the quality and functionality of the refined oil, although previous studies by Warner & Dunlap [3] and Warner [14], found expeller-processing improved the frying characteristics of refined soybean oil as compared with solvent-extracted oil. Thus, oil processing practices can influence the frying performance and functionality of canola oil and warrants further research. 

The objective of this work was to investigate the effects that different extraction practices have on the frying life of canola oil. Canola oils were sampled over two production seasons and used to fry potato chips. The frying life and other quality indices as affected by canola oil type (extraction practice) are discussed. 

## 2. Materials and Methods 

Canola oil samples were supplied by processors in Australia. Fresh cut potato chips (10 mm) were purchased from a local processor (Ezy Fresh Processing, Wagga Wagga, Australia). Fatty acid methyl ester (FAME) and internal (tridecanoic acid, C13:0) standards, standards of α- and γ-tocopherols, DPPH (2,2-diphenyl-1-picrylhydrazyl) and p-anisidine were purchased from Sigma-Aldrich (Castle Hill, NSW, Australia). All other reagents were of analytical grade.

### 2.1. Canola Oil Sampling 

**Figure 1:**
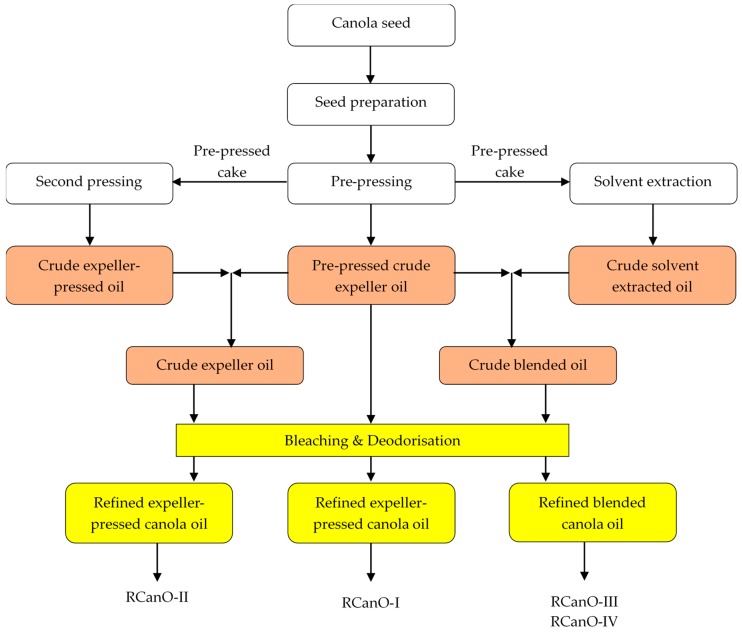
Flow chart showing main processing streams for the different canola oils.

Refined canola oil samples were obtained from Australian oil processing plants undertaking both seed crushing and crude oil refining. Oil samples were from the 2016/2017 and 2017/2018 production seasons and included two refined expeller-pressed (RCanO-I and RCanO-II) and two refined expeller-pressed and solvent-extracted blended (RCanO-III and RCanO-IV) canola oils (Figure 1). Samples were collected within 10 days of manufacture, purged with nitrogen gas and stored at <8 °C. Canola oil samples were used as supplied by processors with no added tocopherols. 

### 2.2. Frying Procedure

Frying was conducted in a 2 × 5 L capacity stainless steel double pan deep fryer (Model FFA2002, Anvil Double Basket Benchtop Fryer, Anvil Axis, Roodepoort, South Africa). Five liters of oil were placed in each pan and heated at 180 ± 5 °C for 7 h each day (1 h preheating + 6 h of frying). For the frying exercise, 500 g of fresh cut potato chips were fried in the hot oil for 8 min every 30 min for a total of 6 h each day (total of 12 frying cycles). Frying was carried out for 3 (2016/2017 samples) or 4 (2017/2018 samples) consecutive days without oil replenishment. The 3 or 4 days of frying occurred because samples from 2016/2017 reached the total polar compounds cut-off mark of 24% on day 3 (deteriorated faster) as compared with the 2017/2018 samples. Fried oils were filtered at the end of frying on day 2 (filtration was performed at about 60 °C to minimize oil losses). At the end of frying each day, 50 mL of oil sample were collected in amber glass bottles and kept at −20 °C for further analyses. 

### 2.3. Assessing Frying Oil Quality

The free fatty acids (FFA) content as oleic acid, peroxide value (PV), and p-anisidine value of oil samples were assessed as per American Oil Chemists’ Society official methods of analysis [15], with minor variations to suit testing conditions. 

### 2.4. Determination of Fatty Acids Composition

Fatty acids composition was determined as fatty acid methyl esters using a capillary gas chromatograph (GC) (7890A GC System, Agilent Technologies, Mulgrave, Australia) equipped with a Zebron ZB-FAME GC Cap column (30 m × 0.2 mm × 0.2 µm; Phenomenex, Lane Cove West, Australia) and a flame ionisation detector (FID) detector. The carrier gas was nitrogen with a flow rate of 1 mL/min and a split ratio 1:100. The injector and detector temperatures were set to 250 °C and 260 °C, respectively; oven temperature was maintained at 100 °C for 2 min, and then increased at a rate of 5 °C/min to 240 °C. The total analysis time was 35 min. The sample was prepared by weighing about 100 mg of canola oil into screw-capped glass tubes, followed by the addition of 500 µL of tridecanoic acid (50 mg/mL in hexane) as internal standard, and the preparation was then diluted with 1.5 mL of hexane. The mixture was vortexed for 30 s and then 200 µL of 2 M methanolic KOH was added and vortexed again for 60 s. The mixture was allowed to stand for 30 min and then centrifuged at 3000 *g* for 5 min. The supernatant was transferred into chromatographic vials for GC analysis and 1 µL injected onto the column. The fatty acid methyl esters were identified using authentic FAME standard and quantified by the internal standard method [16]. 

### 2.5. Determination of Tocopherols Content 

Tocopherols were determined by normal phase HPLC under isocratic condition using a Phenomenex Luna silica column (150 mm × 4.6 mm, 3 µm, 100 A) fitted with a silica 4 × 3.0 mm Security Guard cartridge (Phenomenex Australia Pty, Sydney, Australia) on an HPLC system. Canola oil (260–750 mg, depending on the oil sample, weights of aged oil were increased to improve quantification) was dissolved in 10 mL hexane and filtered through 0.45 µm syringe filters into amber HPLC vials, with 10 µL being injected onto the column. The mobile phase was hexane: isopropanol (99.5:0.5 v/v) and the flow rate was 1.0 mL/min (total run time was 20 min). A Varian Star INERT 9012 binary pump was used with a Varian ProStar Model 410 autosampler. Tocopherols were determined using a Prominence Fluorescence detector (RF—20A XS, Shimadzu, Melbourne, Australia) with excitation wavelength, 290 nm and emission wavelength, 330 nm. Five-point calibration curves were constructed in the ranges of 0.50–6.00 mg/L and 1.00–15.00 mg/L for α- and γ-tocopherols, respectively, and used to quantify tocopherols. 

### 2.6. Determination of Total Polar Compounds (TPC)

The TPC of canola oils was measured using a Testo 270 oil tester (Testo Pty Ltd., Croydon South, Australia). Briefly, the Testo 270 tester was calibrated each day (prior to start of frying) against a calibration oil supplied by the manufacturer (heated to 50 °C). After each frying cycle and removal of the basket of fried chips, the oil was allowed to equilibrate for 5 min before taking TPC measurements. Measurements were conducted as per manufacturer’s instructions. The TPC was measured before the start of frying each day and then at every 60 min of frying (i.e., after fry # 2, 4, 6, 8, 10, and 12). Fryers were visually divided into two portions and readings were taken of the upper and lower portions, with the average result used as the TPC value. 

### 2.7. DPPH Radical Scavenging Antioxidant Activity

The total radical scavenging activity of the oil samples was analyzed using the DPPH radical-scavenging assay as described by Tuberoso et al. [17], with slight modifications. The oil sample (500 µL) was mixed with ethyl acetate (500 µL) and vortexed for 30 sec. Then, 150 µL of the oil solution was reacted with 3350 µL of DPPH solution (0.04 mM in ethyl acetate) in a test tube. The mixture was immediately vortexed for 10 sec, incubated in the dark for 20 min, and the final absorbance was measured at 517 nm. The ethyl acetate DPPH solution was used as a control. The percentage of DPPH scavenging activity was calculated as the antioxidant activity. 

### 2.8. Statistical Analysis 

All experiments were performed in duplicate, and the means ± standard deviations are reported. Analysis of variance was performed (Statgraphics^®^ Centurion 18, StatPoint Technologies, Inc., Warrenton, VA, USA) and mean separation performed using Fisher’s least significant difference (LSD) test (*p <* 0.05).

## 3. Results and Discussion 

### 3.1. Initial Oil Quality

The initial quality of the oil is important to establish the oxidation state before frying and is presented in Table 1 and Table 2 (0 cycle). The free fatty acids (FFA) content of the unfried canola oils ranged from 0.04% to 0.06% (*p* > 0.05), peroxide and p-anisidine values from 0.35 to 0.80 mEq O_2_/kg oil and 0.84 to 1.85, respectively, for both production seasons (*p* < 0.05). The FFA values were consistent with those reported by Aladedunye and Przybylski [18], however, the canola oils used in the current study had lower peroxide values. For both seasons, the RCanO-II samples recorded highest peroxide values, whereas the RCanO-III recorded the highest p-anisidine values. Both the peroxide and p-anisidine values assessed the pre-frying oxidation state of oils. The peroxide value is indicative of formation of hydroperoxides (primary oxidation) and the p-anisidine value is indicative of the decomposition of hydroperoxides (secondary oxidation). Hydroperoxides decompose to carbonyl compounds of secondary oxidation, however, no apparent correlation was found for peroxide and p-anisidine values, probably due to the intrinsic differences among the canola oil types. All canola oils, however, were of good quality and suitable for frying, as peroxide values were below the guidelines (10 mEq O_2_/kg oil) set by Codex for refined vegetable oils [19].

Since β- and δ-tocopherols are typically low in canola oils, only α- and γ-tocopherols were analyzed for total tocopherol concentration. Tocopherol concentration ranged from 610–682 mg/kg and 640–742 mg/kg from the 2016/2017 and 2017/2018 seasons, respectively. The 2017/2018 samples had higher tocopherol concentrations as compared with the 2016/2017 samples (*p <* 0.05), which can increase frying life [14]. The tocopherol concentrations reported in this study were higher than those previously reported for refined canola oils by Mba et al. [7], i.e., 182.4 mg/kg and Ghazani et al. [1], i.e., 326.8 mg/kg, but were comparable to values reported by Aladedunye and Przybylski [18]. 

Fatty acid compositions were typical of canola oil (Table 2, 0 cycle) as follows: oleic acid (C18:1) ranged from 59.7%–64.5%, linoleic acid (C18:2) ranged from 17.5%–20.8%, and linolenic acid (C18:3) ranged from 9.26%–10.3%, for both seasons. No statistically significant differences were found for fatty acids for both season (*p > 0.05*). The RCanO-II had the highest C18:1 content, but the lowest C18:2 and C18:3 contents for both seasons, with comparable compositions for RCanO-I, RCanO-III, and RCanO-IV. The contents of C18:1, C18:2, and C18:3 correlated with their MUFA, PUFA, and iodine values. Overall, only marginal differences existed between the canola oil types, which is expected to have little impact on their frying characteristics. 

The pre-frying tocopherol concentrations and fatty acid compositions were indicative of seed clustering zones. Typically, processed seeds are sourced within a 200 km to 300 km radius, hence, the type seeds processed, their fatty acid compositions and tocopherol concentrations are dependent on the growing area and season, and the nature and pattern of seed aggregation (varieties and proportions). These can impact the seed and refined oil qualities, and hence their food applications. Again, different processing practices (extraction technique) and refining capabilities of the processing plants may affect the refined oil quality, as well as the retention of minor bioactive components, for example, tocopherols. Therefore, more research into the effects of seed clustering zones, growing seasons, oil extraction, and refining practices on canola oil functionality is warranted. 

### 3.2. Total Polar Compounds (TPC)

**Figure 2:**
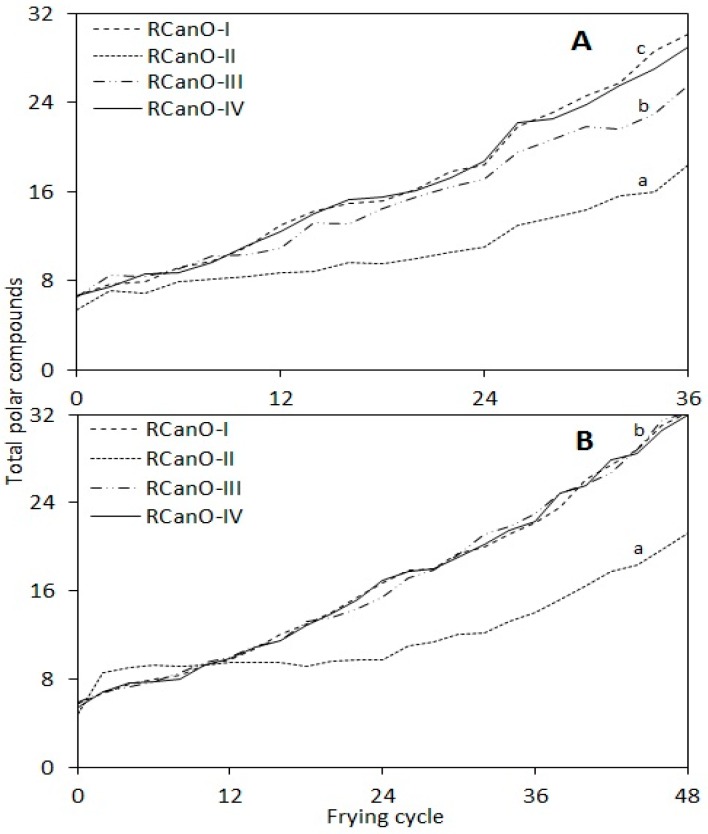
Changes in total polar compounds of canola oils during 36 and 48 frying cycles: (**A**) 2016/2017 (*n* = 36) and (**B**) 2017/2018 (*n* = 48) production seasons. Different letters show means that are statistically significant from each other (*p <* 0.05).

The measurement of TPC presents an accurate evaluation of oil deterioration during frying [20,21] and has been used to measure quality changes in other foods such as table olives [22]. Consequently, the oil was considered degraded when the TPC value reached 24% [21,23], which is the threshold for the European Union [21], and widely accepted globally. The TPC of canola oils during frying were measured every 60 min and are shown in Figure 2. TPC was monitored for 36 (Figure 2A) and 48 (Figure 2B) frying cycles for the 2016/2017 and 2017/2018 samples, respectively. This occurred because the 2016/2017 samples exhibited faster rates of deterioration as compared with the 2017/2018 samples. For all samples, TPC increased as the number of frying cycles increased, which is indicative of oil deterioration (*p <* 0.05). The trend in TPC evolution was characteristic of the oil type and was consistent for both seasons. For the 2016/2017 samples, RCanO-II exhibited the greatest stability (*p* < 0.05) with TPC only rising from 5.4% to 18.4% after 36 frying cycles in contrast to 6.5% to 25.5%, 6.6% to 29.0%, and 6.6% to 30.1% for RCanO-III, RCanO-IV, and RCanO-I, respectively. A similar trend was found for the 2017/2018 oil samples. Overall, RCanO-I and RCanO-IV exhibited the shortest frying life from 2016/2017 (*p <* 0.05) but had similar frying life to RCanO-III from 2017/2018. 

In Figure 2B, the TPC for the 2017/2018 RCanO-II, initially increased (4.8% to 8.6%) during the first two cycles and then remained nearly constant for the next 24 cycles (i.e., 14 h of heating). Most likely, the complexity of reactions occurring in the oil may suggest a re-adjustment phase as the oil was initially heated. Such reactions could have resulted in increased vaporization of volatiles initially present in the oil, which were detected by the TPC probe, and also the possible formation of other compounds that could have improved the stability of RCanO-II and increased the frying life. Overall, RCanO-II resisted changes in TPC (*p <* 0.05), especially, for the first 24 cycles, indicating lesser deterioration as compared with the other oil types. For example, at end of the 24th cycle, the absolute change in TPC was 5.6% and 5.0% for RCanO-II as compared to 11.6% and 10.6% for RCanO-I, 11.0% and 9.5% for RCanO-III, and 12.2% and 11.6% for RCanO-IV from 2016/2017 and 2017/2018, respectively. 

The absolute change in TPC for each oil type was consistent for both seasons and highlighted differences in the frying properties of the different canola oil types. This may be attributed to the intrinsic quality differences in the canola oil types as determined by processing practices employed by the different processors. A study comparing the frying life of two refined soybean oil by Warner and Dunlap [3] found no differences in the TPC of the two oils, however, it should be noted that the two oils had important differences prior to frying that may have confounded the results. For example, one oil had significantly lower peroxide and TPC values (*p <* 0.05) and contained 200 ppm TBHQ (a potent synthetic antioxidant used in fats and oils) as compared with the other oil [3]. In the current study, RCanO-II exhibited longer frying life as compared to RCanO-I, RCanO-IV, and RCanO-I (*p <* 0.05), suggesting that oil stability may have been influenced by the different processing practices. Moreover, there have been limited studies [3,14] that compared the frying characteristics of refined oils obtained by different processes, although significant work has been done on crude vegetable oils to understand differences in their physicochemical characteristics. This warrants further studies. 

### 3.3. Free Fatty Acids (FFA) Content 

**Figure 3:**
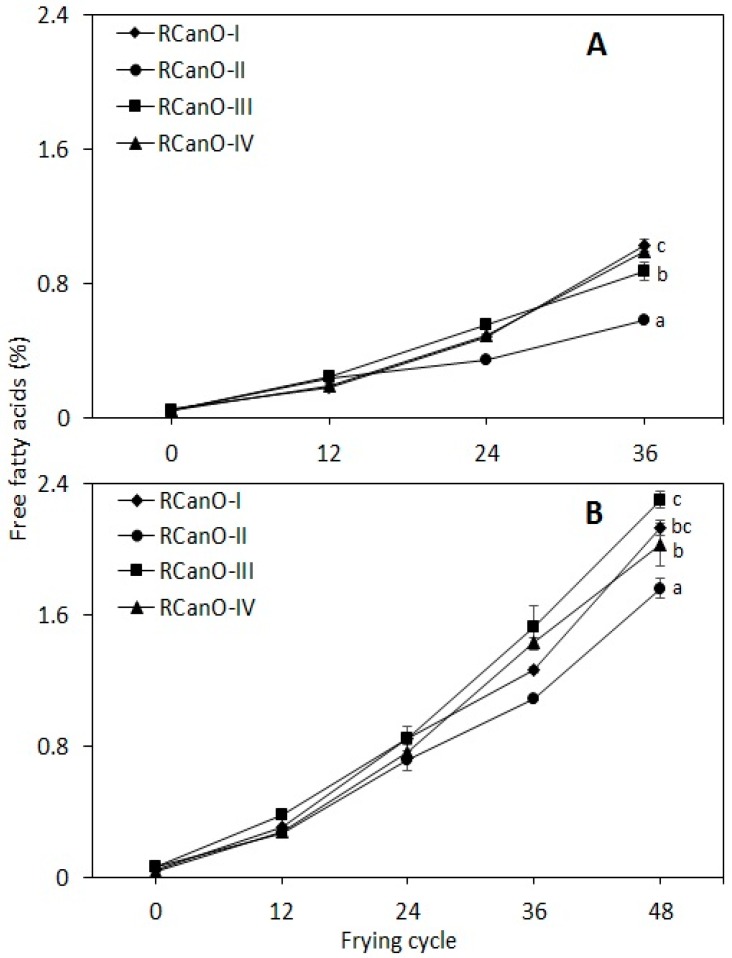
Formation of free fatty acids in canola oils during 36 and 48 frying cycles: (**A**) 2016/2017 (*n* = 36) and (**B**) 2017/2018 (*n* = 48) production seasons. Different letters show means that are statistically significant from each other (*p <* 0.05).

The evolution in the FFA content of edible oils is indicative of hydrolytic rancidity caused by hydrolysis of ester bonds of triglycerides and as degradation products from oxidized triglycerides [4,24], and contributes to off-flavors in fried oils and foods. In the current study, frying conditions (temperature, time, and oil: food ratio) were kept constant, hence, it was expected that changes in the oils (initial moisture content was below detection) would result from their intrinsic properties and their susceptibility to hydrolysis and thermolysis. RCanO-II showed greater stability against hydrolysis and thermolysis of the triglycerides, as evidenced by the least changes in the FFA for both seasons (Figure 3). RCanO-I and RCanO-IV (2016/2017), and RCanO-III (2017/2018) recorded the greatest degree of hydrolysis with FFA increasing from 0.05% to 1.1% and 0.04% to 1.0%, and 0.06% to 2.3%, respectively. The differences in FFA formation with frying can be related to the physicochemical characteristics of the individual oils and their response to moisture and heating during frying. 

The FFA values were higher than values reported for high-oleic low-linolenic rapeseed oil, high-oleic sunflower oil, mid-oleic sunflower oil, low-linolenic soybean oil, and palm oleic [24,25,26]. Differences in the oil types and frying conditions most likely accounted for the variations and, also, frozen par-fried potato chips were used in these studies, whereas fresh potato chips were used in the current study. The high moisture content of freshly cut chips, up to 80% [27], as compared with par-fried potato chips, is likely to lead to greater hydrolysis, because breakage of triglycerides to give FFA is facilitated in the presence of water and steam [28].

Comparatively, oil samples from 2017/2018 exhibited higher FFA with frying than 2016/2017, suggesting that the 2017/2018 samples may have a greater tendency to go rancid as compared with the 2016/2017 samples. Conversely, the 2017/2018 canola samples recorded the least changes in fatty acids composition (Section 3.4). Oils from the two seasons showed different degradation reactions. The 2016/2017 samples showed greater polymerization (Figure 2) whereas the 2017/2018 samples showed greater hydrolysis (Figure 3). Thus, the different reactions occurring in the oil at any particular time dictated the proportions of the various compounds formed during frying. Irrespective of the oil type, FFA were well correlated with the frying time and were individually fitted to a second (2nd) order polynomial curve (r^2^ = 0.97 to 0.99) (data not shown). A linear relationship existed particularly at less than three days (<36 cycles) of frying but not at greater than three days of frying (longer, >36 frying cycles). Thus, the trend in FFA development with frying was a function of the oil type and the frying cycle. 

### 3.4. Fatty Acid Composition 

Monitoring changes in the relative proportions of fatty acids is used to assess the thermo-oxidation of oils during deep-frying [4]. The change in the fatty acid composition of oils is presented in Table 2 (36th and 48th cycle). The minor fatty acids were unchanged during frying, possibly due to their low proportion in the oils (<0.25%, data not shown). Conversely, the relative proportions of the major fatty acids, C16:0, C18:0, and C18:1 increased in response to a decrease in C18:2 and C18:3, which is consistent with previous studies [4,18]. 

**Figure 4:**
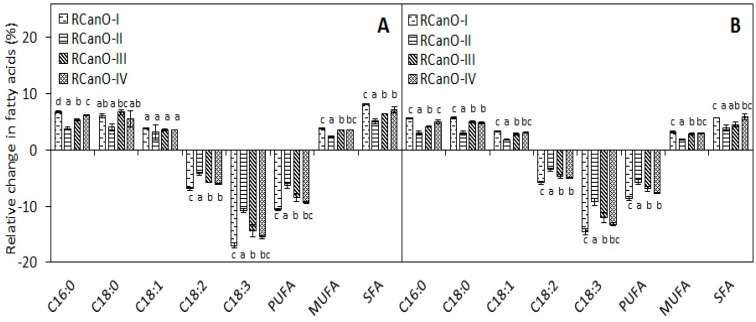
Changes in selected fatty acids in canola oils during 36 and 48 frying cycles: (**A**) 2016/2017 (*n* = 36) and (**B**) 2017/2018 (*n* = 48) production seasons; PUFA, polyunsaturated fatty acid; MUFA, monounsaturated fatty acid; SFA, saturated fatty acid; and IV, iodine value. Different letters show means that are statistically significant from each other (*p* < 0.05).

The percentage change in the major fatty acids is presented in Figure 4. Linoleic acid (C18:2) decreased by magnitudes of 4.2% to 6.9% and 3.5% to 5.8%, and linolenic acid (C18:3) decreased by 10.8% to 17.0% and 9.2% to 14.6% from 2016/2017 and 2017/2018, respectively. The decrease in the proportions of polyunsaturated fatty acids led to a resultant increase in the monounsaturated fatty acids by 3.1% to 3.8% (2016/2017) and 1.8% to 3.3% (2017/2018). Aladedunye and Przybylski [18] also reported that linoleic and linolenic acid decreased by 5.7% and 18.5%, respectively, and oleic acid increased by 1.5% after 28 h of frying with canola oil. The change in fatty acids composition was consistent with previous reports [8,29]. The decrease in the C18:2 and C18:3 can be explained by the following: (i) thermo-oxidation to form hydroperoxides and subsequent degradation into secondary oxidation products (Section 3.6) and (ii) polymerization reactions between fatty acids (Figure 2). Thus, during frying, degradation of polyunsaturated fatty acids resulted in an increase in the proportions of the saturated and monounsaturated fatty acids, and a decrease in the iodine value (Table 2 and Figure 4). Iodine value measures the degree of unsaturation in oils and a decrease during frying is indicative of thermo-oxidative reactions [30]. Irrespective of the season, the RCanO-II recorded the least change in fatty acids, followed by RCanO-III and RCanO-IV, and then RCanO-I (*p <* 0.05) which indicated greater stability of this canola oil as compared with the others. 

### 3.5. Tocopherol Retention

**Figure 5:**
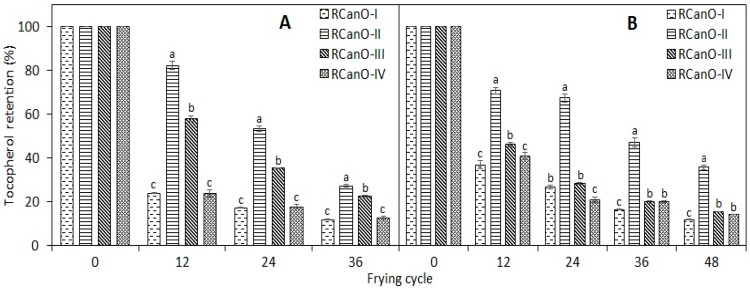
Tocopherols retention in canola oils during 36 (**A**): 2016/2017 production season and 48 (**B**): 2017/2018 production season, frying cycles. Different letters show means that are statistically significant from each other (*p* < 0.05).

Figure 5 shows the retention in tocopherols during 36 and 48 frying cycles with canola oils. RCanO-II had the highest retention in tocopherols as compared with RCanO-I, RCanO-III, and RCanO-IV (*p* < 0.05) from both seasons. RCanO-III had higher retention than the RCanO-I and RCanO-IV for the 2016/2017 samples (*p* < 0.05) but had a similar retention for the 2017/2018 samples. Comparatively, higher retention of tocopherols was found for the 2017/2018 samples than for the 2016/2017 samples. Higher tocopherol concentrations (Table 1) implies the greater likelihood of higher retention during frying. 

Tocopherols provide stability against auto- and thermo-oxidative changes in oils. Consequently, higher tocopherol retention by oils can lead to longer frying life and greater oil stability. In the current study, RCanO-II presented the highest retention in tocopherols (Figure 5) for both 2016/2017 (27.3%) and 2017/2018 (35.7%), which paralleled an increased frying life as measured by changes in TPC (Figure 2), fatty acids composition (Figure 4 and Table 2), p-anisidine value (Section 3.6), and the antioxidant activity (Section 3.7). Przybylski et al. [20] found that soybean oil showed faster tocopherol degradation and also had faster development of polar compounds during deep-frying, highlighting the protective effect of tocopherols on oils during frying. Thus, the ability of the RCanO-II oils to retain more tocopherols contributed to their increased frying life. 

The tocopherol concentration in oil is influenced by the growing season, climatic conditions during growing and harvesting, seed storage and handling [31], seed variety, and crude oil extraction, as well as oil storage, blending, and refining practices. It is estimated that about 25% of tocopherols are lost by the end of the deodorization step during refining of rapeseed oil [32]. Thus, modern refining practices are capable of retaining sufficient amounts of tocopherols in oil, with differences in refined oil tocopherols being influenced by the aforementioned factors, especially the oil extraction practices [1], and the seed cluster zones, and might also explain the differences observed in frying with the different oil types. 

The 2017/2018 RCanO-II oil retained the highest tocopherols partly because it had the highest initial tocopherol (Table 1), and hence retained the most during frying. Canola oils from 2016/2017, contained similar amounts of tocopherols (Table 1), with differences in tocopherol retention most likely due to variations in the quality of the unfried oil, and their susceptibility to the frying condition and associated chemical reactions. As shown in Table 1, although RCanO-II had the highest initial peroxide values for both seasons, this did not impact adversely on the frying characteristics, suggesting factors other than peroxide value might have contributed to the longer frying life. A study by Warner and Dunlap [4,25] highlighted the contribution of Maillard reaction products (MRPs) to the oxidative stability of expeller-pressed soybean oil during frying. Higher temperatures (typically from 90 to 140 °C) of expeller processing may favor the formation of MRPs which have been shown to confer high oxidative stability on oils [33]. The presence of some of these lipophilic MRPs [33], in the expeller-pressed RCanO-II sample, may have protected tocopherols from heat and oxidative deterioration, and led to greater retention during frying, and therefore warrants further studies. Other factors including the presence of pro-oxidative compounds, and post-refining handling and storage may initiate oxidative reactions that may affect oil stability and lead to variability in the frying life. Again, processing conditions (e.g., heating and reaction time) during oil extraction and refining can lead to diverse chemical reactions in oils, which may affect tocopherol availability, stability, reactivity, and functionality. 

### 3.6. p-Anisidine Value

**Figure 6:**
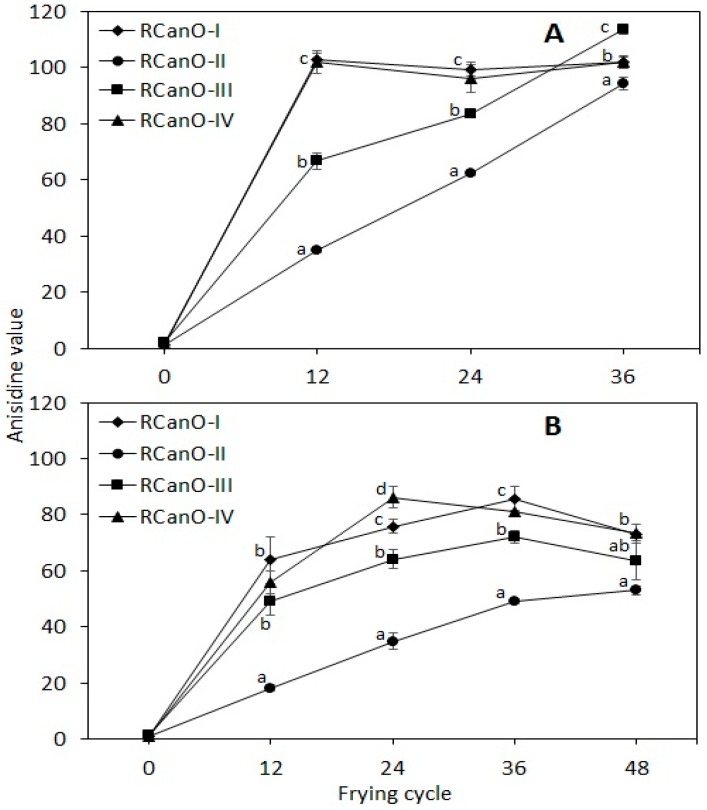
Formation of nonvolatile carbonyls (as p-anisidine) during 36 and 48 frying cycles: (**A**) 2016/2017 (*n* = 36) and (**B**) 2017/2018 (*n* = 48) production seasons. Different letters show means that are statistically significant from each other (*p* < 0.05).

The evolution in p-anisidine value as an index of secondary oxidation was characteristic of each oil (Figure 6). The anisidine value measures the amount of nonvolatile carbonyl compounds [4,25], principally 2-alkenals and 2,4-alkadienals [4], and provides information on the thermo-oxidative state of frying oils. On the one hand, the p-Anisidine values for 2016/2017 RCanO-II increased linearly from 1.29 to 94.47 (Figure 6A). The p-anisidine values for RCanO-I and RCanO-IV increased steeply from 1.23 to 102.95 and 1.57 to 102.00 (12 frying cycles), respectively, and remained constant for the rest of the frying cycles. On the other hand, RCanO-III gradually increased from 1.85 to 113.52 after 36 frying cycles. Unlike the 2016/2017 season, the p-anisidine values for the 2017/2018 RCanO-I (0.85 to 85.53), RCanO-III (1.30 to 71.99), and RCanO-IV (1.30 to 71.99) samples increased gradually and then declined. 

RCanO-I and RCanO-IV recorded higher p-anisidine values as compared with RCanO-III (*p* < 0.05), indicating a more extensive degradation of unsaturated fatty acids [25] in RCanO-I and RCanO-IV, which is consistent with data presented in Figure 4. Similar to 2016/2017, the 2017/2018 RCanO-II increased in a linear fashion especially for the first 36 cycles. Consistent with other quality indices, RCanO-II had lower p-anisidine values (*p* < 0.05) indicating lower oxidation, and hence greater stability and longer frying life. The p-anisidine values were comparable to those reported for high oleic sunflower oil [25], refined olive pomace oil, and olive pomace and coconut oil blends [4], but were lower than those reported for rapeseed oil after 16 h of frying [30].

The stability of the oils followed a similar trend for both seasons, however the samples from 2016/2017 had higher p-anisidine values compared to 2017/2018 (Figure 5B and Figure 6A), underscoring a greater stability of oil samples from 2017/2018. Greater stability of the 2017/2018 samples is also evidenced by the longer frying life (48 cycles), lower rates in TPC evolution (Figure 2), and the least change in the fatty acid composition (Table 2 and Figure 4). The p-anisidine values for each oil correlated with the tocopherol retention during frying (r^2^ = −0.85 to −0.96, calculated using the combined data for both seasons for each oil), with higher tocopherol retention associated with lower p-anisidine values and longer frying life. 

Moreover, the time dependence in the evolution of p-anisidine with frying depended on the combined effect of the oil type (processing practice, expeller or expeller-solvent blends) and the frying cycle. Except for RCanO-II, the p-anisidine values for RCanO-I, RCanO-III, and RCanO-IV were poorly correlated with frying time, which is consistent with a previous report by Aladedunye and Przybylski [18], for canola oil fried at 185 °C and 215 °C. These variations underscore the inherent quality differences between the different oils, which influenced the type and rate of reactions occurring in the oils during frying. 

Frying studies have typically been conducted on oils from different plant sources with marked differences in their fatty acids composition [8], blends of different oils [29], same oil type but different frying conditions [18], and regular and modified oils [20]. In the current study, canola oils with similar fatty acid compositions and obtained by different processing practices across two production seasons were compared, which has highlighted the impact of different processing practices on the frying life of canola oil. 

### 3.7. Radical Scavenging Antioxidant Activity

**Figure 7:**
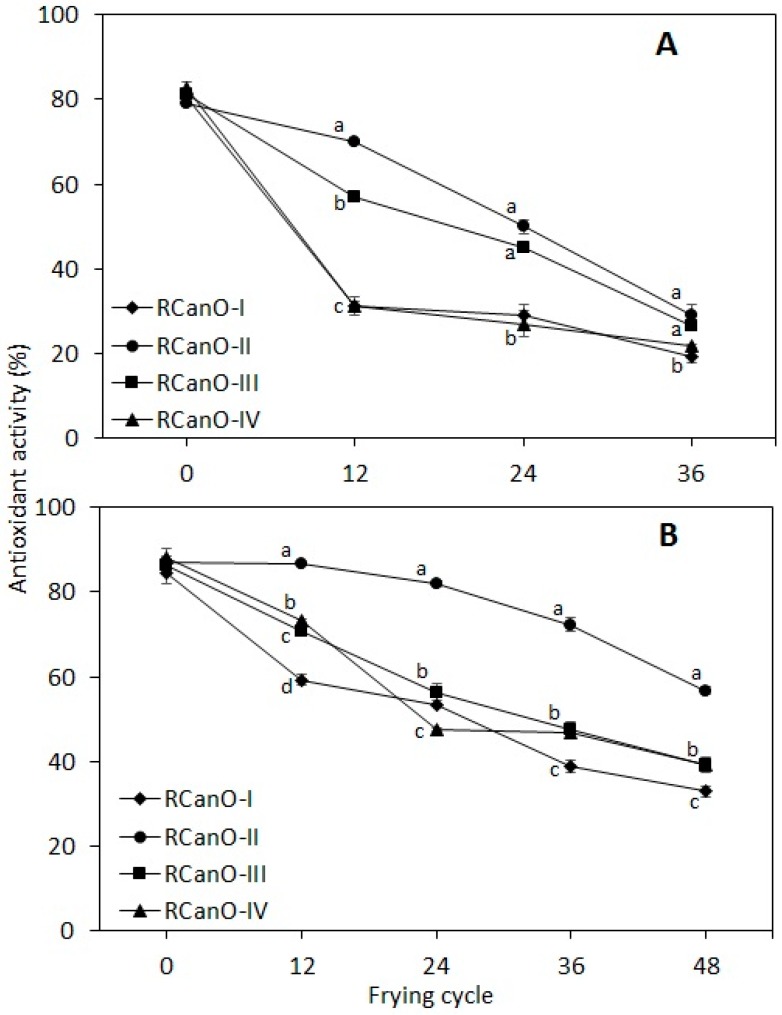
Antioxidant activity of canola oils during 36 and 48 frying cycles: (**A**) 2016/2017 (*n* = 36) and (**B**) 2017/2018 (*n* = 48) production seasons. Different letters show means that are statistically significant from each other (*p* < 0.05).

The antioxidant activity of the oils is shown in Figure 7. As expected, the radical scavenging activity of all oils declined significantly with progressive frying (*p* < 0.05), which is consistent with previous studies [5,34]. Before frying, oils from each season showed similar antioxidant activity, i.e., 78.9% to 81.1% from the 2016/2017 season and 84.6% to 87.00% from the 2017/2018 season (*p* > 0.05). Overall, RCanO-II showed the highest antioxidant activity as compared with RCanO-I, RCanO-III, and RCanO-IV from both seasons (*p* < 0.05). The trend in antioxidant activity correlated with tocopherol retention (Figure 5) with the highest tocopherol retention displaying the highest antioxidant activity and the longest frying life (Figure 2). For example, RCanO-II retained more tocopherols (Figure 5) during frying and also exhibited the highest antioxidant activity. The converse was true for the RCanO-I, RCanO-III, and RCanO-IV. 

The decrease in antioxidant activity with frying was correlated with tocopherol concentration (r^2^ = 0.99, 0.94) and the p-anisidine value (r^2^ = −0.97, −0.90) for 2016/2017 samples and 2017/2018 samples, respectively. Likewise, the antioxidant activity for each sample was correlated with the TPC (r^2^ = −0.98 to −0.82) during progressive frying. This highlighted the important role tocopherols play in the antioxidative properties of refined oils and the effect of antioxidants on the thermo-oxidative stability of oils during frying, which was consistent with previous studies [34].

## 4. Conclusions

The quality changes in canola oils during frying were investigated. The results demonstrated functional differences between the different canola oil types during frying, with some canola oils demonstrating superior frying life as compared with others. Overall, oil samples from the 2017/2018 production season had longer frying life than those from the 2016/2017 production season, highlighting the seasonal variations between canola oils from both seasons. Tocopherol retention influenced the frying life of the oils and stability of tocopherols with frying was found to be more important than the quantity present. Regardless, the effect on frying characteristics was influenced by the processing (extraction) practices of the different processors. This influenced the quality of refined oils (e.g., composition and concentration of minor components), and hence their frying characteristics. The marked difference in frying life of the different oils indicates that future research into processing practices and conditions on oil functionality is warranted.

## Figures and Tables

**Table 1:** Initial oil quality of canola oils from the 2016/2017 and 2017/2018 production seasons.

Oil type	RCanO-I		RCanO-II		RCanO-III		RCanO-IV	
Season	2016/2017	2017/2018	2016/2017	2017/2018	2016/2017	2017/2018	2016/2017	2017/2018
Free fatty acids (FFA) %	0.05 ± 0.004	0.04 ± 0.002	0.06 ± 0.001	0.06 ± 0.001	0.05 ± 0.000	0.06 ± 0.001	0.05 ± 0.002	0.04 ± 0.000
Peroxide vale (PV) (mEq O_2_/kg Oil)	0.39 ± 0.001 ^a,k^	0.53 ± 0.03 ^b,l^	0.79 ± 0.004 ^b,k^	0.80 ± 0.05 ^c,k^	0.42 ± 0.03 ^a,l^	0.35 ± 0.001 ^a,k^	0.58 ± 0.02 ^a,k^	0.54 ± 0.04 ^a,k^
*p*-Anisidine Value	1.18 ± 0.02 ^a,l^	0.84 ± 0.04 ^a,k^	1.29 ± 0.04 ^ab,k^	1.15 ± 0.04 ^b,k^	1.85 ± 0.01 ^c,l^	1.27 ± 0.06 ^b,k^	1.55 ± 0.15 ^b,l^	0.91 ± 0.11 ^a,k^
Tocopherols (mg/kg)	626 ± 1.80 ^b,k^	652 ± 9.69 ^c,k^	616 ± 4.88 ^c,l^	742 ± 6.61 ^a,k^	610 ± 1.22 ^c,l^	640 ± 4.98 ^c,k^	682 ± 4.99 ^a,k^	685 ± 8.99 ^b,k^

Different letters show means that are statistically significant from each other (*p <* 0.05), ^abc^ compare different oil types within same production season, and ^kl^ compares same oil type for the two seasons.

**Table 2:** Fatty acids composition of canola oils from the 2016/2017 and 2017/2018 production seasons.

Season	Oil type	Fatty Acids	C16:0	C18:0	C18:1	C18:2	C18:3	MUFA	PUFA	SFA	IV
2016/2017	RCanO-I	0 cycle	4.05 ± 0.01	2.07 ± 0.00	62.4 ± 0.00	18.7 ± 0.02	10.1 ± 0.01	63.9 ± 0.00	28.8 ± 0.03	7.30 ± 0.02	113.5 ± 0.05
		36th cycle	4.32 ± 0.01	2.19 ± 0.01	64.8 ± 0.06	17.4 ± 0.03	8.36 ± 0.03	66.4 ± 0.06	25.8 ± 0.06	7.87 ± 0.00	108.9 ± 0.08
	RCanO-II	0 cycle	4.11 ± 0.00	1.98 ± 0.00	63.0 ± 0.01	18.3 ± 0.00	9.78 ± 0.01	64.6 ± 0.01	28.1 ± 0.01	7.29 ± 0.01	112.6 ± 0.03
		36th cycle	4.27 ± 0.01	2.06 ± 0.01	64.5 ± 0.07	17.6 ± 0.06	8.72 ± 0.06	66.1 ± 0.08	26.3 ± 0.12	7.62 ± 0.04	109.8 ± 0.19
	RCanO-III	0 cycle	4.65 ± 0.00	1.86 ± 0.01	59.7 ± 0.04	20.8 ± 0.01	10.3 ± 0.07	61.3 ± 0.05	31.0 ± 0.07	7.69 ± 0.02	115.3 ± 0.14
		36th cycle	4.90 ± 0.01	1.99 ± 0.00	61.8 ± 0.08	19.6 ± 0.05	8.80 ± 0.05	63.4 ± 0.09	28.4 ± 0.11	8.19 ± 0.02	111.3 ± 0.16
	RCanO-IV	0 cycle	4.17 ± 0.01	2.03 ± 0.01	61.3 ± 0.02	19.4 ± 0.02	10.3 ± 0.01	62.9 ± 0.02	29.7 ± 0.01	7.39 ± 0.01	114.4 ± 0.01
		36th cycle	4.43 ± 0.00	2.15 ± 0.02	63.5 ± 0.04	18.2 ± 0.04	8.69 ± 0.03	65.2 ± 0.04	26.9 ± 0.07	7.92 ± 0.02	110 ± 0.02
2017/2018	RCanO-I	0 cycle	4.03 ± 0.01	1.90 ± 0.00	62.3 ± 0.01	19.6 ± 0.01	9.69 ± 0.01	63.8 ± 0.01	29.2 ± 0.00	7.00 ± 0.00	113.8 ± 0.01
		48th cycle	4.24 ± 0.02	1.96 ± 0.01	64.3 ± 0.19	18.5 ± 0.12	8.32 ± 0.13	65.8 ± 0.20	26.8 ± 0.24	7.40 ± 0.00	110.1 ± 0.36
	RCanO-II	0 cycle	4.07 ± 0.01	1.85 ± 0.00	64.5 ± 0.01	17.5 ± 0.01	9.26 ± 0.01	66.1 ± 0.02	26.8 ± 0.03	7.10 ± 0.00	111.2 ± 0.04
		48th cycle	4.18 ± 0.01	1.90 ± 0.01	65.7 ± 0.11	16.9 ± 0.07	8.44 ± 0.10	67.3 ± 0.13	25.4 ± 0.17	7.35 ± 0.07	109.1 ± 0.27
	RCanO-III	0 cycle	4.19 ± 0.01	1.86 ± 0.00	61.0 ± 0.02	20.1 ± 0.01	9.63 ± 0.01	62.5 ± 0.01	30.3 ± 0.01	7.20 ± 0.00	114.6 ± 0.01
		48th cycle	4.37 ± 0.03	1.95 ± 0.01	62.8 ± 0.22	19.7 ± 0.13	8.42 ± 0.15	64.4 ± 0.22	28.1 ± 0.28	7.55 ± 0.07	111.2 ± 0.41
	RCanO-IV	0 cycle	4.15 ± 0.01	1.84 ± 0.01	61.2 ± 0.01	20.3 ± 0.01	9.74 ± 0.02	62.8 ± 0.02	30.1 ± 0.03	7.15 ± 0.02	114.5 ± 0.06
		48th cycle	4.36 ± 0.01	1.92 ± 0.01	63.1 ± 0.12	19.3 ± 0.04	8.47 ± 0.09	64.7 ± 0.10	27.8 ± 0.13	7.54 ± 0.03	111.0 ± 0.22

PUFA, polyunsaturated fatty acid; MUFA, monounsaturated fatty acid; SFA, saturated fatty acid; and IV, iodine value.
